# Time-Series Transcriptomic Analysis Reveals the Molecular Profiles of Diapause Termination Induced by Long Photoperiods and High Temperature in *Chilo suppressalis*

**DOI:** 10.3390/ijms232012322

**Published:** 2022-10-14

**Authors:** Haibo Bao, Hui Zhu, Peihan Yu, Guanghua Luo, Ru Zhang, Qian Yue, Jichao Fang

**Affiliations:** 1 Jiangsu Key Laboratory for Food and Safety-State Key Laboratory Cultivation Base of Ministry of Science and Technology, Institute of Plant Protection, Jiangsu Academy of Agricultural Sciences, Nanjing 210014, China; 2 Institute of Agricultural Resources and Environment, Jiangsu Academy of Agricultural Sciences, Nanjing 210014, China

**Keywords:** *Chilo suppressalis*, diapause termination, molecular profiles, time series, transcriptomes

## Abstract

Survival and adaptation to seasonal changes are challenging for insects. Many temperate insects such as the rice stem borer (*Chilo suppressalis*) overcome the adverse situation by entering diapause, wherein development changes dynamically occur and metabolic activity is suppressed. The photoperiod and temperature act as major environmental stimuli of diapause. However, the physiological and molecular mechanisms that interpret the ecologically relevant environmental cues in ontogenetic development during diapause termination are poorly understood. Here, we used genome-wide high-throughput RNA-sequencing to examine the patterns of gene expression during diapause termination in *C*. *suppressalis*. Major shifts in biological processes and pathways including metabolism, environmental information transmission, and endocrine signalling were observed across diapause termination based on over-representation analysis, short time-series expression miner, and gene set enrichment analysis. Many new pathways were identified in diapause termination including circadian rhythm, MAPK signalling, Wnt signalling, and Ras signalling, together with previously reported pathways including ecdysteroid, juvenile hormone, and insulin/insulin-like signalling. Our results show that convergent biological processes and molecular pathways of diapause termination were shared across different insect species and provided a comprehensive roadmap to better understand diapause termination in *C. suppressalis*.

## 1. Introduction

The capacity to enter diapause is pervasive in insects which enables them to survive adverse seasonal environments such as harsh winters and synchronise their life cycle with favourable conditions for development and reproduction [1,2]. Diapause is not a cessation of development; instead, it is a dynamic alternate developmental process involving physiologically distinct phases: pre-diapause, diapause, and post-diapause [3]. Furthermore, diapause is a succession of three subphases: initiation, maintenance, and termination [3,4]. Insect diapause can be obligatory, with no external environmental stimuli and occur regardless of environmental conditions, or facultative, wherein specific environmental cues trigger a response and induce diapause [3]. Insects have diverse diapause timings and may occur in the egg, larva or nymph, pupa, or adult stage [4,5]. The photoperiod and temperature are the primary environmental signals for facultative diapause initiation [6]. Especially the photoperiod is the major signal through which most insects anticipate seasonal changes [7].

Environmental signals can also stimulate insects to terminate diapause. This occurs when environmental conditions are favourable with the stimuli passing a critical threshold, allowing insects to continue ontogenetic development [8]. Termination of diapause under field conditions (horotelic termination) is a relatively slow process [9]. In contrast, termination of diapause under laboratory conditions (tachytelic termination) can be rapid [9,10]. Insects can perceive various environmental signals as stimuli for diapause termination. For example, low temperature is a necessary signal to terminate diapause for some insects such as *Chymomyza costata* [4], whereas moisture is more important for other insects [11]. The photoperiod is the most reliable and steady environmental signal amongst signals that seasonally change. Therefore, many insects have evolved to utilise the photoperiod for not only inducing of diapause but also for terminating it [3]. Although not as steady as the photoperiod and temperature, as mentioned earlier, it also plays an important role in the termination of diapause [4,12,13].

The rice stem borer *Chilo suppressalis* is one of the most serious pests of rice in Asia [14] and causes significant annual damage to crop yield [15]. *C. suppressalis* larvae are capable of undergoing facultative diapause when the day length is shorter than the critical duration in autumn [16], even though the average temperature is still above 27 °C in the field [16]. Diapausing *C. suppressalis* larvae are able to endure cold and lack of food, which enables them to survive upcoming harsh winters [17]. Laboratory studies showed that *C. suppressalis* can be induced to enter diapause by a short photoperiod, and night length is a key component for initiating diapause [16,18,19]. In contrast, diapause can be terminated when field-collected *C. suppressalis* larvae (from the winter period) are exposed to long photoperiods (light:dark, 16:8) and high temperatures (15–30 °C) in the laboratory [16].

Many studies have focused on revealing the physiological and molecular mechanisms of diapause under field or laboratory conditions. Transcriptomic analysis of field-collected pre-diapausing *C. suppressalis* larvae revealed that pathways involving neuroactive ligand–receptor interaction, lysosomes, and glycerolipid metabolism were associated with pre-diapause [20]. Comparative transcriptomic analysis of 11 insect species revealed that a set of core genes involved in circadian rhythmicity, insulin signalling, and Wnt signalling were differentially regulated during diapause [21]. However, our current understanding of the molecular mechanisms of diapause termination is unclear.

In this study, diapausing *C. suppressalis* larvae and diapause-terminating larvae induced by long photoperiods (light:dark 16:8) and high temperature (25 °C) were collected at three different time points and their transcriptomes sequenced. Examination of the patterns of gene expression in *C. suppressalis* revealed the biological processes and molecular pathways involved in diapause termination in a panoramic manner based on over-representation analysis, short time-series expression miner, and gene set enrichment analysis.

## 2. Results

### 2.1. Characteristics, Functional Annotation, and Classification of Assembled Transcripts

Transcriptomic changes in *C. suppressalis* larvae during diapause termination were examined in response to long photoperiods (light:dark 16:8) and high temperature (25 °C) at 0, 4, 8, and 12 days (designated as WSG1, WSG2, WSG3, and WSG4, respectively).

A total of 85.75 Gb of clean bases (number of clean reads multiply read length, saved in Gb unit) containing 580.78 million clean reads (reads that passed quality control) was generated from these four groups with 12 samples in all, and each sample contained ≥6.42 Gb of clean bases (43.41 million clean reads) (Table 1). Between 39.93 and 51.21 million clean reads were mapped to the reference genome [22] for each sample, with total map read rates from 84.18 to 93.50%. Approximately 4.22 to 7.14% of total reads were mapped to multiple locations (Table 1). Sequence assembly and functional annotation revealed 25,913 genes, including 14,799 known genes and 11,114 newly detected genes. Together with the transcriptome coverage calculated (Table 1), all results showed that the sequencing had good depth and coverage [23].

### 2.2. Detection of Differentially Expressed Genes (DEGs)

**Figure 1:**
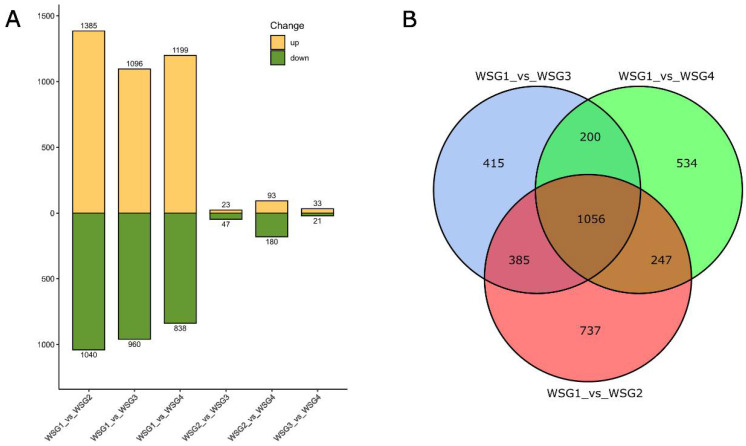
Differently expressed genes (DEGs) of comparison pairs. (**A**) Up- and down-regulated genes in all possible comparisons of diapausing larvae (WSG1) and larvae that were exposed to 4, 8, and 12 days of long day and high temperature conditions in the laboratory (WSG2, WSG3, WSG4 respectively). The numbers on the top and bottom of each end of the bar indicate the quantities of up- and down regulated DEGs. (**B**) Overview of DEG comparisons of WSG1 versus WSG2, WSG1 versus WSG3, and WSG1 versus WSG4. WSG1 was regarded as the control. The numbers within the circles indicate the DEG quantities.

DEGs were identified by comparing the genes in a total of six possible pairs of diapausing *C. suppressalis* sample groups (WSG1) and three other treated sample groups (WSG2, WSG3, WSG4). A total of 3734 genes were differentially expressed in at least one of the six comparisons (Figure 1A). Among them, 2425 DEGs were identified in WSG1_vs_WSG2 (1385 upregulated genes and 1040 downregulated genes). This was far more than the number identified in WSG2_vs_WSG3 (70 genes) and WSG3_vs_WSG4 (54 genes). Meanwhile, 2037 DEGs (1199 upregulated genes and 838 downregulated genes) were identified in WSG1_vs_WSG4, which is close to WSG1_vs_WSG3 (2056 DEGs; 1096 upregulated genes and 960 downregulated genes). There were 1056 shared or common DEGs in the following comparisons: WSG1_vs_WSG2, WSG1_vs_WSG3, and WSG1_vs_WSG4 (Figure 1B), which represented the DEGs between the state of diapause and terminating diapause.

**Figure 2:**
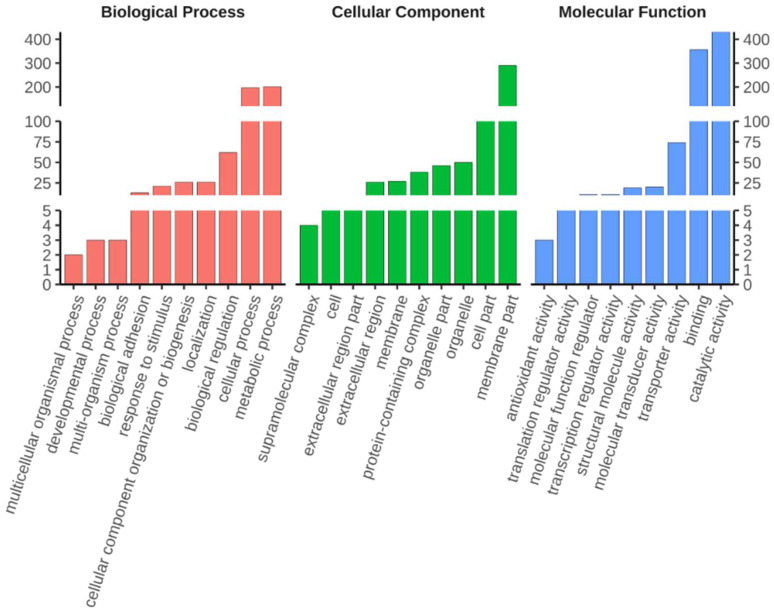
Gene ontology (GO) terms that were significantly enriched in shared DEGs upon diapause termination. The GO terms containing the top ten number of DEGs (with ties) within three GO annotation categories (stated at the top of the image) are shown. The *y*-axis indicates the number of DEGs.

**Figure 3:**
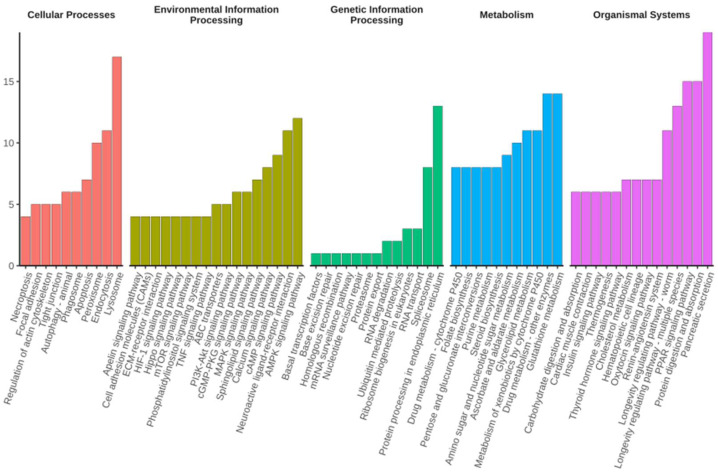
Kyoto Encyclopaedia of Genes and Genomes (KEGG) pathways that were significantly enriched in shared DEGs upon diapause termination. KEGG pathways with the top 10 number of DEGs (with ties) within one KEGG category are shown. The *y*-axis indicates the number of DEGs.

Shared DEGs were classified on the basis of Gene Ontology (GO) and Kyoto Encyclopaedia of Genes and Genomes (KEGG) annotations to reveal the biological processes and pathways associated with diapause termination. GO classification was performed for three major categories: biological process, molecular function, and cellular component. Most DEGs were assigned to metabolic and cellular process, followed by biological regulation in biological processes. Meanwhile, most DEGs were categorised into catalytic activity, binding, and transporter activity in the molecular function group. Finally, most DEGs were classified to membrane- and cell parts in terms of cellular components (Figure 2, Supplementary Table S1). KEGG orthology classification was applied to the shared DEGs in all top categories of the KEGG pathway maps. Lysosomes had the most DEGs in the cellular process category, while the most predominant DEGs were found in the cAMP signalling pathway, neuroactive ligand–receptor interaction, and AMPK signalling pathway in the environmental information-processing category. Protein processing in the endoplasmic reticulum contained the most DEGs for the genetic information processing category, while drug metabolism and pancreatic secretion DEGS were mostly found in metabolism and organismal systems, respectively (Figure 3, Supplementary Table S2). The category of human diseases is not shown in the figure because *C. suppressalis* is an insect.

### 2.3. Short Time-Series Expression Miner

A short time-series expression miner (STEM) was applied to all DEGs from all six possible comparisons of WSG1, WSG2, WSG3, and WSG4 to detect temporal differences in gene expression profiles associated with diapause termination.

**Figure 4:**
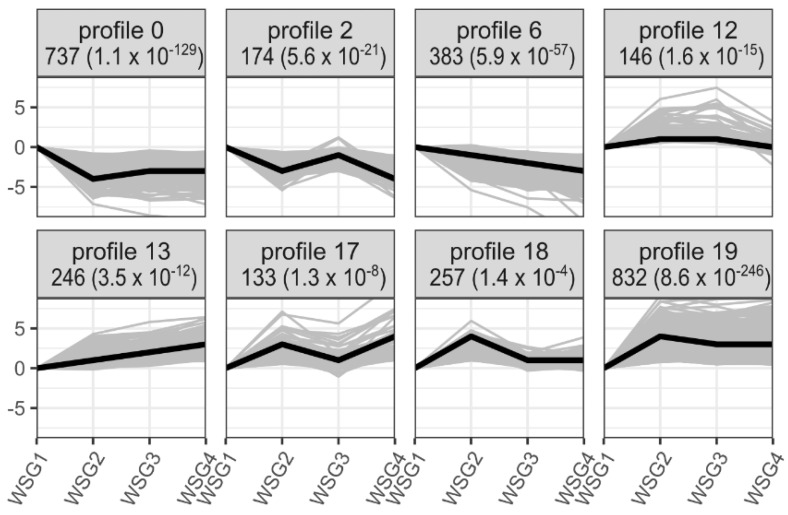
Significant temporal expression patterns of DEGs from all pairwise comparisons of WSG1-WSG4. All DEGs served as input in short time-series expression miner (STEM) analysis. The eight profiles suggesting the expression patterns of clustered genes are significant (*p* < 0.05) are all shown. The number of DEGs for each profile are noted at the top of each panel followed by a *p* value in parentheses. Thin grey lines indicate expression patterns of each individual genes clustered in the profile, while thick black lines represent the expressional trend of each profile. WSG1-WSG4 on the *x*-axis represent 0, 4, 8, and 12 days of treatment, respectively, while the numeric values on the *y*-axis represent normalised expression values.

Twenty profiles were clustered according to gene expression. Eight of these profiles showed significant expressional patterns in the process of diapause termination (*p* < 0.05) (Figure 4). Profiles 0, 2, and 6 contained clusters of genes exhibiting downregulated expression trends, whereas the genes of all other profiles showed upregulated expression trends. Profile 0 (737 genes) and profile 19 (832 genes) clustered more DEGs than the other profiles. Subsequently, GO enrichment analysis was performed on each of these clustered profiles to identify overrepresented biological pathways or processes. Profiles 0, 2, 6, 12, 13, 17, 18, and 19 had significantly enriched GO terms (q value < 0.05) (Table 2). Profile 0 showed significantly enriched genes related to biosynthetic processes (including small molecules, fatty acids, carboxylic acids, and monocarboxylic acids) and catabolic processes (including organic substances and organonitrogen compounds) (Table 2). Profile 19 only showed significantly enriched GO terms in small molecule catabolic processes and extracellular regions.

KEGG enrichment analysis was also implemented to each profile. Profiles 0, 13, 17, 18, and 19 were identified as significant KEGG pathways (q-value < 0.05) (Table 3). Profile 0 showed significantly enriched genes involved in the PPAR signalling pathway, AMPK signalling pathway, Rap1 signalling pathway, GTP-binding proteins, and proteasome. Meanwhile, steroid biosynthesis, insect hormone biosynthesis, pancreatic secretion, and PPAR signalling pathways were enriched in profile 19 (Table 3). The pathways of insulin secretion and neuroactive ligand–receptor interactions were enriched in the other profiles (Table 3).

### 2.4. Changes during the Onset of Diapause Termination

**Figure 5:**
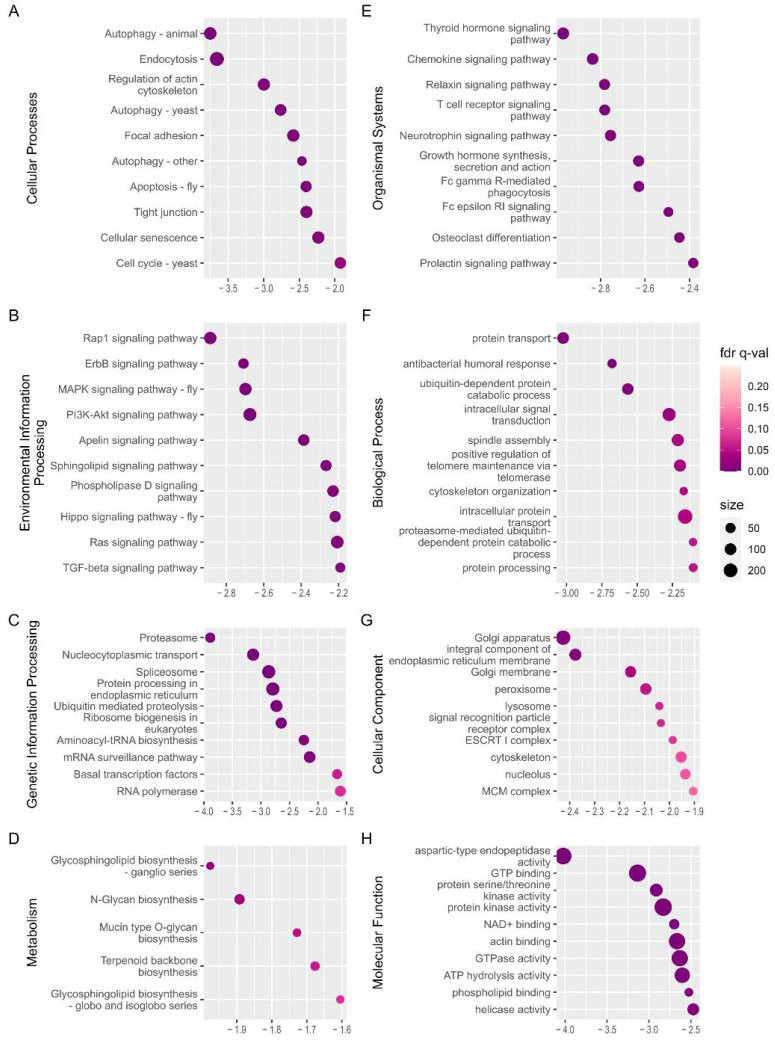
Gene set enrichment analysis (GSEA) of WSG1 versus WSG2. (**A**–**E**) Significantly negatively enriched gene sets of the top categories of KEGG orthology pathways; the *y*-axis shows the definition from the gene set of the KEGG pathway. (**F**–**H**) Significantly enriched gene sets of GO annotation of major categories; the *y*-axis shows the GO terms of the enriched gene set. The normalised enrichment score (NES) is indicated on the *x*-axis in (**A**–**H**). A negative sign in NES demonstrates that gene sets were activated in state of WSG1. The circle size represents the number of genes in each enriched gene set.

Gene set enrichment analysis (GSEA) using GO gene sets and KEGG pathway gene sets was applied to WSG1 vs. WSG2 to reveal the functional interaction within all expressed genes at the onset of diapause termination. There were 148 negatively enriched gene sets (normalised enrichment score (NES) with a minus sign) and 59 positively enriched gene sets (NES with a positive sign) out of the total 431 detected gene sets from the KEGG pathway (Supplementary Table S3). The top 10 negatively enriched KEGG orthology (KO) terms of each category (except Brite hierarchies and human disease) ordered by the absolute value of NES are illustrated in Figure 5. Most of these pathways (such as the Rap1 signalling pathways and MAPK signalling pathway) were also found in KEGG annotations of shared DEGs, which supported and validated the results.

GO gene set analysis showed that 137 out of the 2786 detected gene sets were significantly negatively enriched and 92 positively enriched (Supplementary Table S4). The top 10 negatively enriched GO terms ordered by the absolute value of NES are shown in Figure 5F–H and included protein transport, intracellular signal transduction, and GTP binding. The negative sign of NES demonstrates that the gene sets were activated in state of WSG1.

### 2.5. Changes Occurring in Late-Stage Diapause Termination

The first pupa appeared after 20 d of exposure to long-day photoperiods. Thus, the time point WSG4 (12th day) is likely to be in the late stage of diapause termination. Therefore, the dominant biological processes and physiological characteristics of late-stage diapause termination can be revealed by GSEA analysis applied to WSG1 and WSG4.

**Figure 6:**
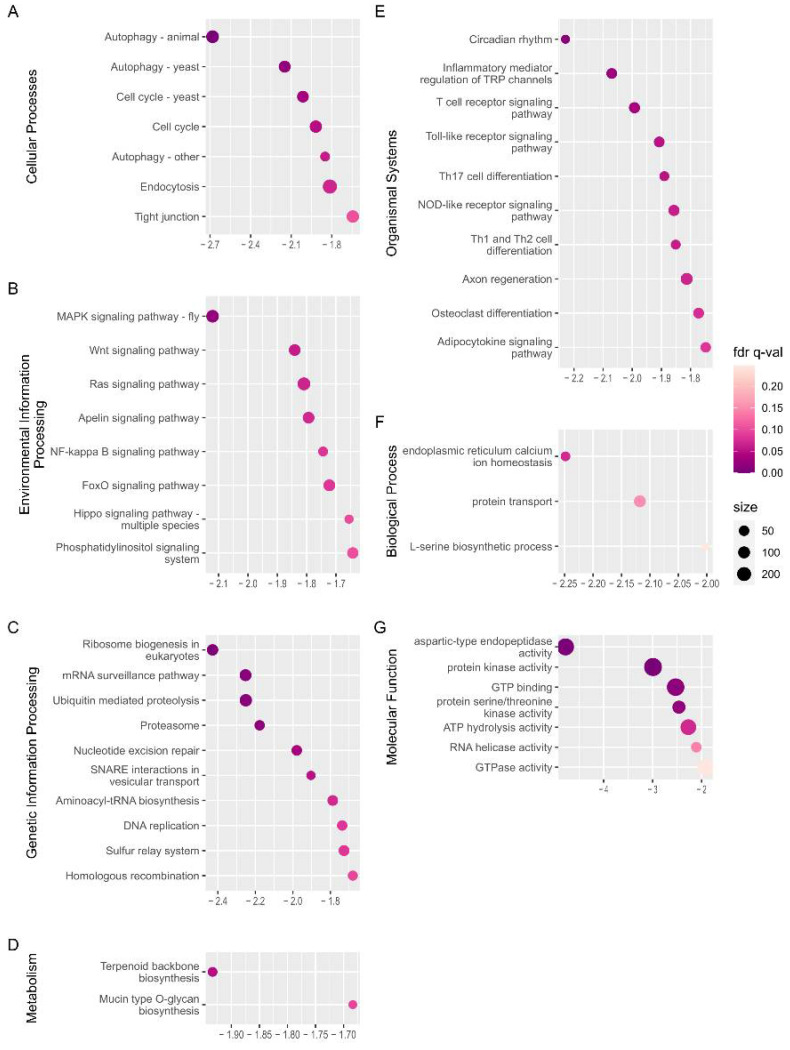
GSEA analysis of WSG1 versus WSG4. (**A**–**E**) Significantly negatively enriched gene set of the top KEGG orthology pathway categories; the *y*-axis shows the definition of the gene set from the KEGG pathway. (**F**,**G**) Significantly negatively enriched gene set of GO annotation from the major domains; the *y*-axis shows the GO terms of the enriched gene set. The NES is indicated on the *x*-axis in (**A**–**G**). A negative sign in NES demonstrates that gene sets were activated in state of WSG1. The circle size represents the number of genes from each enriched gene set.

Seventy-four gene sets out of the total detected KEGG gene sets (431) (Supplementary Table S5) and 11 gene sets out of the total detected GO gene sets (2876) (Supplementary Table S6) were significantly negatively enriched, while these positively enriched KEGG and GO gene sets were 84 and 80, respectively (Supplementary Tables S5 and S6). The top 10 negatively enriched terms of each category for KEGG (except Brite hierarchies and human disease) ordered by the absolute value of NES are illustrated in Figure 6. There were much fewer negatively enriched KEGG gene sets or GO gene sets than those of WSG1 vs. WSG2 (148 and 137, respectively), and no gene set was negatively enriched under the GO category of cellular component. Cellular processes, autophagy, and cell cycle pathways were predominant (Figure 6A). The MAPK signalling pathway, ribosome biogenesis, terpenoid backbone biosynthesis, and circadian rhythm were ranked ahead in other KEGG categories (Figure 6B–E).

### 2.6. Verification of RNA Sequencing Data by Quantitative Polymerase Chain Reaction (qPCR)

**Figure 7:**
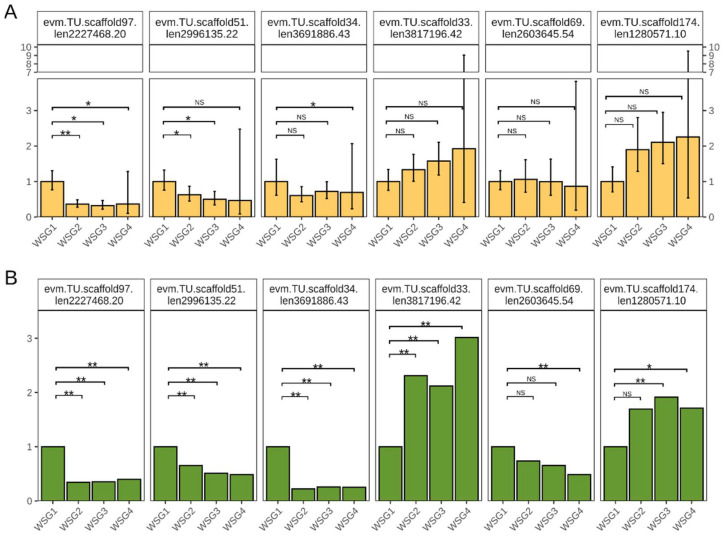
Gene expression pattern verification. (**A**) Relative expression quantification of selected genes by qPCR. Error bars indicate the standard error among biological replicates. NS is not significant, * *p*-adjusted < 0.05, ** *p*-adjusted < 0.01 using Student’s *t*-test and multiple testing corrected with Benjamini–Hochberg method. (**B**) Gene expression fold change of selected genes using WSG1 as a control. Significance was determined by DEseq2, * *p*-adjusted < 0.05, ** *p*-adjusted < 0.01.

The credibility of the RNA sequencing data was confirmed by qPCR. Six genes were selected from the DEGs and their relative expression levels were determined. Four genes were downregulated compared with WSG1 (evm.TU.scaffold97.len2227468.20, evm.TU.scaffold51.len2996135.22, evm.TU.scaffold34.len3691886.43, evm.TU.scaffold69.len2603645.54), whereas two genes were upregulated (evm.TU.scaffold33.len3817196.42, evm.TU.scaffold174.len1280571.10) (Figure 7A). These gene expression profiles were consistent with those of RNA-sequencing (Figure 7B), although the qPCR relative expression levels at WSG4 were highly variable.

## 3. Discussion

*C. suppressalis* larvae exhibit facultative diapause, which is mainly affected by the photoperiod [16]. Larvae enter diapause in autumn (short photoperiods) and terminate it the following spring (long photoperiods co-occur with high temperature) when undisturbed in the field. A state of diapause allows *C. suppressalis* to endure and survive harsh winter conditions and prolonged periods without food to continue self-perpetuating populations when conditions are favourable. This study characterised the gene expression patterns across the developmental time course of diapause termination in *C. suppressalis*. Field-collected larvae were exposed to long photoperiods and high temperatures in the laboratory to terminate diapause, which contrasts with previous studies involving extreme endocrine or pharmacological manipulation. Previous studies mainly focused on comparisons of non-diapausing and diapausing states [20,24,25,26], or clarifying the threshold of environmental stimuli to induce or terminate diapause [16,19,27]. The results of our study provide a roadmap to understand the physiological and molecular mechanisms associated with insect diapause termination, especially in Lepidoptera.

When taking the order of events in diapause termination into account, it is reported that the increasing metabolic rates signal the onset of diapause termination and resumption of development. Studies in *Rhagoletis pomonella* demonstrated that the timing of thousands of DEGs co-occurred with increased metabolism during diapause termination [28], and an increased metabolic rate is a reliable indicator of this event [29]. In other words, differential expression of thousands of genes could be an indicator of the onset of diapause termination. Our study showed that the most differentially expressed genes were found in the comparison of WSG1 vs. WSG2 (Figure 1A). This suggests that the metabolic rates in *C. suppressalis* larvae dramatically increased after exposure to four long and warm days. Changes in the expression of WSG2_vs_WSG3 and WSG3_vs_WSG4 were small compared to WSG1_vs_WSG2 (Figure 1A), indicating that no dramatic change occurred between day 5–12 of the long photoperiod, high temperature treatment. This pattern suggests that *C. suppressalis* terminated diapause within four days of long light and high temperature at the transcriptional level. However, the precise timing of diapause termination in *C. suppressalis* requires further study.

A total of 1056 shared DEGs were identified when WSG2, WSG3, and WSG4 were compared to WSG1 (Figure 1B). These DEGs reflected the difference in gene expression between diapausing and diapause-terminating *C. suppressalis*. KEGG classification of shared DEGs showed that multiple pathways play essential roles in diapause termination (Figure 3). Many differentially expressed KEGG pathways found in *C. suppressalis* diapause termination were also found in *R. pomonella* (Diptera family member that diapauses in pupa), such as mTOR signalling, purine metabolism, and steroid biosynthesis [28]. Metabolites such as glucose, pyruvate, succinate, and malate increased during diapause termination in *Helicoverpa armigera* triggered by injecting 20E [30]. Interestingly, pathways such as carbohydrate metabolism, glycan biosynthesis and metabolism, and pyruvate metabolism were classified as shared DEGs (Figure 3). Enzyme activities associated with glycogen metabolism (such as phosphorylase and glycogen synthetase) were significantly altered during diapause in *C. suppressalis* [31], confirming the credibility of our experimental results. Furthermore, the similarity in diapause termination metabolic processes among different insect species suggests that they may share analogous mechanisms.

GSEA can provide insights into biological mechanisms in gene sets (i.e., KEGG pathways and GO categories) level between two states of interest, particularly that are subtle at the level of individual genes [32]. Accordingly, the onset and late stage of diapause termination of *C. suppressalis* were analysed with GSEA in this study. Since the WSG1 was set as control, negatively enriched pathways and biological processes are these that activated in the state of WSG1 and positively enriched ones are these that activated in the state of WSG2 or WSG4. In other words, negatively enriched pathways and biological processes are functioning to maintain insects in state of diapause. Therefore, they are important to explain the molecular changes of diapause termination.

Time-series analysis confirmed that major gene expression changes occurred in the first period of the time course involving diapause termination, especially profiles 0 and 19, which contained nearly half of the genes analysed (Figure 4). Many GO terms related to biosynthetic and catabolic processes were enriched in these two profiles (Table 2), suggesting consistency in the increasing metabolic rate during the initiation of diapause termination [28].

Extensive research has shown that changes in environmental cues initiate a cascade of endocrine events that control diapause [5,33]. Steroid biosynthesis and insect hormone biosynthesis pathways enriched in profile 19 showed consistency in the endocrine system in response to changes in photoperiod length (Table 3). Moreover, these endocrine events occurred in the early stages of diapause termination (Figure 4).

The ecdysteroid-, juvenile hormone (JH)-, and insulin/insulin-like signalling pathways regulate responses to environmental changes and are involved in diapause in many insects [5,34]. In pre-adult diapause, ecdysteroids (such as 20-hydroxyecdysone in insects) are generally regarded as diapause terminators [5,34]. Meanwhile, JH induces and maintains diapause in *C. suppressalis* [35]. In *Culex pipiens*, JH triggers diapause termination, and JH production is regulated by the insulin/insulin-like signalling pathway and the downstream *FoxO* gene [36]. Insulin-like peptides and its downstream FoxO signalling pathway increase trehalose anabolism and trigger diapause termination in *Antheraea pernyi* [37]. Hydrogen peroxide-induced diapause termination in *Artemia cyst* embryos showed that DEGs are involved in the Hippo- and MAPK signalling pathways [38]. Extracellular signal-regulated kinase (ERK) (a member of the mitogen-activated protein kinase (MAPK) family) is activated during the egg diapause-terminating process in *Locusta migratoria* [39]. ERK/MAPK regulates ecdysteroid and sorbitol metabolism in *Bombyx mori* through the transcription of key enzymes (sorbitol dehydrogenase-2 and EPPase) for embryonic diapause termination [40,41]. These pathways were also identified in this study during *C. suppressalis* diapause termination using STEM- or GSEA analysis. These consistencies validate the reliability of our results and demonstrate the convergence of mechanisms of diapause termination between different insects.

Currently, the links between environmental information such as the photoperiod and endocrine signals are poorly understood [42]. However, numerous studies have indicated that circadian clock genes and their regulators contribute to diapause regulation. A *period* gene knockout line of *B. mori* shows that the GABAeric pathway mediates the photoperiod information to the diapause hormone pathway [43]. Circadian transcription factors vrille and Par domain protein 1 may regulate signalling pathways underlying arrested eggs follicle development and fat accumulation in diapausing *Culex pipiens* females [44]. Circadian genes *period* and *timeless* promote lipid accumulation during diapause preparation in the diapause destined female *Colaphellus bowringi* [45]. N-acetyltransferase is considered a critical conjunction of photoperiodism between the circadian system and endocrine axis in *Antheraea pernyi* [46]. Our GSEA analysis of WSG1 and WSG4 pairs showed that the circadian rhythm pathway (ko04710) was the top enriched gene set (by NES) in the organismal system category (Figure 6), suggesting that this pathway also plays an important role in diapause termination of *C. suppressalis*. It is notable that the circadian rhythm pathway was not enriched in GSEA analysis in the comparison of WSG1_vs_WSG2. This likely indicates that only a few core genes participated in the early process of photoperiod information transmissions. This finding should be validated in future studies.

The identification of candidate genes and signalling pathways for diapause termination in *C. suppressalis* provides a foundation for understanding how environmental cues are transformed into endocrine events that regulate the diapause process. The key genes and pathways may be utilised to disturb the diapause process of *C. suppressalis* as a green strategy to control this pest insect. For instance, if *C. suppressalis* terminates diapause in an untimely manner, it cannot survive harsh conditions. Generally, exploring the mechanisms of diapause regulation will facilitate comparative research on regulatory factors conserved across insects [21]. Our results show that convergent biological processes and molecular pathways are shared across different insect species, although the specific genes regulating diapause termination may not be the same.

## 4. Materials and Methods

### 4.1. Insects and Collection of Samples

Diapausing *C. suppressalis* larvae were collected from rice straw left at the paddy side from Huai’an, Jiangsu, in January 2021. A random sample of diapausing larvae was immediately collected and designated WSG1. The remaining diapausing larvae were reared in the laboratory at 25 ± 1 °C under long photoperiod light:dark cycles (16:8 h) and a relative humidity of 70 ± 5% to terminate diapause. One sample group was collected every four days (at 9:00 a.m.) and a total of three groups (WSG2–WSG4) were collected. The remaining larvae were reared until pupation stage. The interval between the last sampling day and the day where the pupa was observed was noted. Each sample group contained three replicates, and each replicate consisted of four *C. suppressalis* larvae. All collected samples were frozen in liquid nitrogen and stored at −80 °C.

### 4.2. RNA Extraction, Library Construction, and Illumina Sequencing

RNA extraction, library construction, and sequencing were performed by Majorbio Bio-Pharm Biotechnology Co., Ltd. (Shanghai, China). Briefly, total RNA was extracted from stored *C. suppressalis* samples using TRIzol^®^ reagent according to the manufacturer’s instructions (Invitrogen, Thermo Fisher Scientific, Waltham, MA, USA). The sequencing library was prepared using high-quality RNA (OD260/280 = 1.8–2.2, OD260/230 ≥ 2.0, RIN ≥ 6.5, 28S:18S ≥ 1.0) following the TruSeqTM RNA sample preparation kit (Illumina, San Diego, CA, USA) using 1 μg of the total RNA. Sequencing was performed with an Illumina NovaSeq 6000 instrument (2 × 150 bp read length) according to the manufacturer’s recommendations.

### 4.3. Read Mapping and Gene Functional Annotation

Raw reads were trimmed and filtered with SeqPrep (v. 1.2, https://github.com/jstjohn/SeqPrep (accessed on 29 October 2021)) and Sickle (v. 1.33, https://github.com/najoshi/sickle (accessed on 29 October 2021)) using default parameters. Then, clean reads were aligned to the reference genome in orientation mode using HISAT2 (v. 2.1.0, http://tophat.cbcb.umd.edu/ (accessed on 29 October 2021)) software, and the mapped reads were assembled using StringTie (v. 2.1.2, https://ccb.jhu.edu/software/stringtie/ (accessed on 29 October 2021)) software to obtain a full-length Unigene library. Gene functional annotation was performed using the following databases: non-redundant (NR), Swiss-Prot, Pfam, EggNOG, GO, and KEGG. To estimate transcriptome coverage, the number of annotated genes in each sample were divided by the number of annotated coding genes in the genome of *C. suppressalis*.

### 4.4. Differential Expression Gene Analysis

DEGs were identified between sample groups by calculating the expression level of each transcript according to the transcript per million (TPM) mapped reads method. RNA-sequencing by expectation-maximization (RSEM, v. 1.3.3, http://deweylab.biostat.wisc.edu/rsem/ (accessed on 29 October 2021)) was used to quantify gene abundance. The R package DESeq2 (differential expression analysis for sequence count data 2, v. 1.24.0) was used to analyse the DEGs. Genes with an absolute value fold-change ≥2 and *p*-adjusted value < 0.05 (using the Benjamini–Hochberg method for multiple testing correction) were considered differently expressed.

### 4.5. GO and KEGG Enrichment Analysis

GO and KEGG enrichment analyses were performed based on over-representation analysis (ORA) with the R package ClusterProfilter (v. 4.2.1) [47]. GO terms and KEGG pathways with *p*-adjusted values < 0.05 were used for subsequent analyses. Multiple testing was performed with the Benjamini–Hochberg method.

### 4.6. Short Time-Series Expression Miner

Short time-series expression miner (STEM) software (v. 1.3.11) was employed to cluster all DEGs from pairwise comparisons of WSG1-WSG4 to detect temporal gene expression patterns. Gene expression values were transformed into log-normalised data which served as input data. STEM analysis was performed using the STEM clustering method with default settings, except that the maximum unit change in the model profiles between time points was changed to 4. Each expression profile represented a gene set associated with the time-course of diapause termination in *C. suppressalis*. Gene sets with a *p* value < 0.05 were considered significant.

### 4.7. Gene Set Enrichment Analysis

Gene set enrichment analysis using GO gene sets and KEGG pathway gene sets were applied to WSG1 vs. WSG2 and WSG1 vs. WSG4, respectively, wherein WSG1 served as a control when calculating the log2 fold change of gene expression. This was performed to reveal the functional interaction within all expressed genes in different time courses involved in diapause termination. The WSG2 time point reflects transcriptional changes in the initiation of diapause termination, while the WSG4 time point shows transcriptional changes in the posterior process of diapause termination.

Genes in paired comparisons were ranked according to log2 transformed expression fold change, and the ranked gene list was analysed using GSEA package (v. 4.2.1, https://www.gsea-msigdb.org/gsea/index.jsp (accessed on 6 January 2022)). The analysis was performed using the GSEAPreranked tool with default settings except that the enrichment statistic was set to classic and min size was set to 1. KEGG and GO annotations of the *C. suppressalis* transcriptome were used as enrichment gene set data. Enriched gene sets with a nominal *p*-value < 0.05, FDR q-value < 0.25, and absolute value of normalised enrichment score (NES) > 1 were considered significant.

### 4.8. qPCR Verification

Six randomly selected DEGs were evaluated by qPCR to verify their expression levels in RNA sequencing. Specific primers (Supplementary Table S7) were designed using Primer3 (https://bioinfo.ut.ee/primer3-0.4.0/ (accessed on 21 February 2022 )). The qPCR template was prepared from the RNA used for transcriptomic sequencing. Total RNA (1000 ng) was reverse-transcribed to cDNA using PrimeScript^TM^ RT master mix (Perfect Real Time) (TaKaRa, Kusatsu, Japan). Each qPCR reaction was prepared as follows: 10 μL TB Green premix EX Taq II (Tli RNaseH plus) (TaKaRa), 2 μL cDNA template, 7.2 μL ddH_2_O, 0.4 μL forward primer (10 μM), and 0.4 μL reverse primer (10 μM). Each gene was analysed using three biological replicates and each biological replicate had at least three technical replicates. The reactions were carried out using a program that consisted of an initial heat activation step of 95 °C for 30 s, followed by 40 cycles of 95 °C for 5 s and 60 °C for 30 s in a LightCycler 480 II (Roche, Basel, Switzerland). Relative quantification was performed using the 2^−ΔΔCt^ method [48] with *histone 3* as a reference gene [49]. The relative expression levels of genes were compared using Student’s *t*-test instead of the multiple comparison test across all four time-points since all of these DEGs were obtained by pairwise comparison of the control (WSG1) and treatment (WSG2, WSG3, WSG4, respectively). The *t*-test *p*-values were corrected with the Benjamini–Hochberg method for multiple testing.

## 5. Conclusions

Our results provide a panoramic view of the molecular mechanisms of diapause termination in *C. suppressalis* in a time-series manner. The identified genes and molecular pathways associated with diapause termination validate the convergent molecular mechanisms of different insects. The new candidate genes and molecular pathways identified in this study should help integrate the understanding of the mechanisms of diapause termination.

## Figures and Tables

**Table 1:** Quality control and mapped results of sequencing data.

Group	Sample	Clean Bases	Q30 (%)	Clean Reads	Total Mapped (%)	Multiple Mapped (%)	Intergenic Mapped (%)	Transcriptome Coverage (%)
WSG1	WS6	8.24 × 10^9^	94.43	55,656,460	92.01	4.22	17.78	0.80
	WS7	6.77 × 10^9^	94.31	45,797,804	91.92	4.22	16.62	0.80
	WS8	6.42 × 10^9^	94.66	43,410,018	91.99	7.14	16.84	0.83
WSG2	WS10	8.07 × 10^9^	94.33	54,579,676	92.69	6.34	16.24	0.84
	WS11	7.06 × 10^9^	94.18	47,661,440	92.79	5.30	16.16	0.85
	WS12	7.02 × 10^9^	94.27	47,537,308	92.00	6.04	15.63	0.84
WSG3	WS14	6.77 × 10^9^	94.19	45,865,908	92.74	4.80	15.65	0.80
	WS15	7.37 × 10^9^	94.52	49,850,256	93.24	5.14	15.55	0.83
	WS16	6.88 × 10^9^	93.82	46,589,114	92.35	6.32	16.76	0.81
WSG4	WS18	6.57 × 10^9^	94.68	44,441,840	93.50	7.02	12.48	0.80
	WS19	6.82 × 10^9^	95.33	47,035,356	92.27	5.14	16.15	0.84
	WS20	7.77 × 10^9^	95.33	52,353,480	84.18	4.53	15.98	0.83

**Table 2:** GO enrichment of significant profiles from the STEM analysis.

Profiles	Ontology	ID	GO Term	*p* Value	q Value	No. of DEGs
profile 0	BP	GO:0046394	carboxylic acid biosynthetic process	9.5965 × 10^−6^	0.00276	12
profile 0	BP	GO:0016053	organic acid biosynthetic process	1.2252 × 10^−5^	0.00276	12
profile 0	BP	GO:1901575	organic substance catabolic process	3.6778 × 10^−5^	0.0055232	27
profile 0	BP	GO:0009056	catabolic process	6.0569 × 10^−5^	0.00597364	28
profile 0	BP	GO:1901565	organonitrogen compound catabolic process	6.6296 × 10^−5^	0.00597364	18
profile 0	BP	GO:0044283	small molecule biosynthetic process	0.0001319	0.00990415	13
profile 0	BP	GO:0006633	fatty acid biosynthetic process	0.00039385	0.02218004	7
profile 0	BP	GO:0072330	monocarboxylic acid biosynthetic process	0.00039385	0.02218004	7
profile 0	CC	GO:0005838	proteasome regulatory particle	0.00027949	0.02667635	4
profile 0	CC	GO:0022624	proteasome accessory complex	0.00040875	0.02667635	4
profile 6	MF	GO:0004190	aspartic-type endopeptidase activity	2.5896 × 10^−16^	2.5623 × 10^−14^	30
profile 6	MF	GO:0070001	aspartic-type peptidase activity	2.5896 × 10^−16^	2.5623 × 10^−14^	30
profile 12	BP	GO:0006790	sulfur compound metabolic process	0.00027544	0.04494025	5
profile 12	CC	GO:0045211	postsynaptic membrane	0.00074514	0.01464137	3
profile 12	CC	GO:0097060	synaptic membrane	0.00074514	0.01464137	3
profile 12	CC	GO:0098794	postsynapse	0.00074514	0.01464137	3
profile 12	CC	GO:0098590	plasma membrane region	0.00134392	0.01980516	3
profile 12	MF	GO:0004521	endoribonuclease activity	4.1869 × 10^−5^	0.00351926	12
profile 12	MF	GO:0004540	ribonuclease activity	6.1345 × 10^−5^	0.00351926	12
profile 12	MF	GO:0004523	RNA-DNA hybrid ribonuclease activity	0.0001295	0.00388602	11
profile 12	MF	GO:0016891	endoribonuclease activity, producing 5′-phosphomonoesters	0.00015406	0.00388602	11
profile 12	MF	GO:0016893	endonuclease activity, active with either ribo- or deoxyribonucleic acids and producing 5′-phosphomonoesters	0.00016934	0.00388602	11
profile 12	MF	GO:0046983	protein dimerization activity	0.00104623	0.02000677	9
profile 12	MF	GO:0004970	ionotropic glutamate receptor activity	0.00153387	0.02514167	3
profile 12	MF	GO:0008066	glutamate receptor activity	0.00179574	0.02575468	3
profile 12	MF	GO:0004020	adenylylsulfate kinase activity	0.00311503	0.02749299	3
profile 12	MF	GO:0004779	sulfate adenylyltransferase activity	0.00311503	0.02749299	3
profile 12	MF	GO:0004781	sulfate adenylyltransferase (ATP) activity	0.00311503	0.02749299	3
profile 12	MF	GO:0022824	transmitter-gated ion channel activity	0.00311503	0.02749299	3
profile 12	MF	GO:0022835	transmitter-gated channel activity	0.00311503	0.02749299	3
profile 12	MF	GO:0030594	neurotransmitter receptor activity	0.00372919	0.03056257	3
profile 12	MF	GO:0070566	adenylyltransferase activity	0.00490717	0.03753554	3
profile 12	MF	GO:0019239	deaminase activity	0.00574998	0.04123341	2
profile 13	CC	GO:0015935	small ribosomal subunit	0.00019127	0.01026822	5
profile 13	CC	GO:0005840	ribosome	0.00072952	0.01457127	9
profile 13	CC	GO:0044391	ribosomal subunit	0.00081428	0.01457127	5
profile 13	MF	GO:0008238	exopeptidase activity	5.0636 × 10^−8^	6.5027 × 10^−6^	14
profile 13	MF	GO:0004180	carboxypeptidase activity	5.3045 × 10^−5^	0.00340608	7
profile 13	MF	GO:0004177	aminopeptidase activity	0.00010002	0.00364202	7
profile 13	MF	GO:0008237	metallopeptidase activity	0.00013246	0.00364202	15
profile 13	MF	GO:0071949	FAD binding	0.0001418	0.00364202	5
profile 13	MF	GO:0004181	metallocarboxypeptidase activity	0.00034004	0.00727815	5
profile 13	MF	GO:0004568	chitinase activity	0.00119377	0.02190071	3
profile 13	MF	GO:0008235	metalloexopeptidase activity	0.0015807	0.02537435	5
profile 13	MF	GO:0003735	structural constituent of ribosome	0.00216091	0.03083403	8
profile 17	CC	GO:0005576	extracellular region	1.2344 × 10^−5^	0.00058472	12
profile 18	BP	GO:0007018	microtubule-based movement	6.5018 × 10^−7^	0.00012497	8
profile 18	BP	GO:0006928	movement of cell or subcellular component	1.046 × 10^−6^	0.00012497	9
profile 18	BP	GO:0007017	microtubule-based process	0.00013787	0.01098121	9
profile 18	CC	GO:0030286	dynein complex	0.00010304	0.00650806	5
profile 18	CC	GO:0005875	microtubule associated complex	0.0003687	0.00949006	5
profile 18	CC	GO:0005929	cilium	0.00045078	0.00949006	5
profile 18	CC	GO:0042995	cell projection	0.00082612	0.01043518	5
profile 18	CC	GO:0120025	plasma membrane bounded cell projection	0.00082612	0.01043518	5
profile 18	CC	GO:0031966	mitochondrial membrane	0.00129926	0.01367644	6
profile 18	CC	GO:0005740	mitochondrial envelope	0.00195022	0.01759593	6
profile 18	CC	GO:0005743	mitochondrial inner membrane	0.00262559	0.01920079	5
profile 18	CC	GO:0019866	organelle inner membrane	0.00273611	0.01920079	5
profile 18	CC	GO:0005746	mitochondrial respirasome	0.00350664	0.02214723	2
profile 18	CC	GO:0031967	organelle envelope	0.00442094	0.02326809	7
profile 18	CC	GO:0031975	envelope	0.00442094	0.02326809	7
profile 18	CC	GO:0015630	microtubule cytoskeleton	0.00777081	0.03775292	6
profile 18	CC	GO:0005739	mitochondrion	0.00912603	0.04117007	8
profile 18	MF	GO:0003777	microtubule motor activity	5.0355 × 10^−5^	0.00704968	6
profile 19	BP	GO:0044282	small molecule catabolic process	4.1507 × 10^−5^	0.01769514	9
profile 19	CC	GO:0005576	extracellular region	3.5328 × 10^−5^	0.00547585	31

BP, biological process; CC, cellular component; MF, molecular function.

**Table 3:** KEGG enrichment of STEM analysis significant profiles.

Profile	ID	Ko Term	First Category	Second Category	*p* Value	q Value	No. of DEGs
profile 0	ko04514	Cell adhesion molecules	Environmental Information Processing	Signalling molecules and interaction	3.0638 × 10^−5^	0.00870763	8
profile 0	ko03050	Proteasome	Genetic Information Processing	Folding, sorting and degradation	0.00023851	0.0201342	8
profile 0	ko03320	PPAR signalling pathway	Organismal Systems	Endocrine system	0.00027629	0.0201342	9
profile 0	ko04031	GTP-binding proteins	Brite Hierarchies	Protein families: signalling and cellular processes	0.00036265	0.0201342	11
profile 0	ko04611	Platelet activation	Organismal Systems	Immune system	0.00044042	0.0201342	10
profile 0	ko04137	Mitophagy–animal	Cellular Processes	Transport and catabolism	0.0004959	0.0201342	8
profile 0	ko03051	Proteasome	Brite Hierarchies	Protein families: genetic information processing	0.0011115	0.03556506	9
profile 0	ko04361	Axon regeneration	Organismal Systems	Development and regeneration	0.00117699	0.03556506	11
profile 0	ko04145	Phagosome	Cellular Processes	Transport and catabolism	0.00138363	0.03556506	11
profile 0	ko04152	AMPK signalling pathway	Environmental Information Processing	Signal transduction	0.00140832	0.03556506	12
profile 0	ko04141	Protein processing in endoplasmic reticulum	Genetic Information Processing	Folding, sorting and degradation	0.00158249	0.03556506	15
profile 0	ko04212	Longevity regulating pathway–worm	Organismal Systems	Aging	0.00162677	0.03556506	12
profile 0	ko04091	Lectins	Brite Hierarchies	Protein families: signalling and cellular processes	0.00178477	0.03623219	4
profile 0	ko04810	Regulation of actin cytoskeleton	Cellular Processes	Cell motility	0.0021477	0.03814997	12
profile 0	ko04015	Rap1 signalling pathway	Environmental Information Processing	Signal transduction	0.00279784	0.04417641	12
profile 0	ko04510	Focal adhesion	Cellular Processes	Cellular community - eukaryotes	0.00279784	0.04417641	12
profile 13	ko01002	Peptidases and inhibitors	Brite Hierarchies	Protein families: metabolism	2.222 × 10^−8^	2.9003 × 10^−6^	23
profile 13	ko00100	Steroid biosynthesis	Metabolism	Lipid metabolism	6.4478 × 10^−6^	0.0004208	5
profile 13	ko04090	CD molecules	Brite Hierarchies	Protein families: signalling and cellular processes	9.9102 × 10^−6^	0.00043118	10
profile 13	ko04640	Hematopoietic cell lineage	Organismal Systems	Immune system	1.547 × 10^−5^	0.00050482	5
profile 13	ko03010	Ribosome	Genetic Information Processing	Translation	5.1402 × 10^−5^	0.00130969	9
profile 13	ko04614	Renin-angiotensin system	Organismal Systems	Endocrine system	8.0183 × 10^−5^	0.00130969	5
profile 13	ko04612	Antigen processing and presentation	Organismal Systems	Immune system	0.00010464	0.00136578	5
profile 13	ko04979	Cholesterol metabolism	Organismal Systems	Digestive system	0.00018713	0.00222049	6
profile 13	ko04972	Pancreatic secretion	Organismal Systems	Digestive system	0.0002252	0.00244956	8
profile 13	ko03011	Ribosome	Brite Hierarchies	Protein families: genetic information processing	0.00036607	0.0036755	9
profile 13	ko00983	Drug metabolism–other enzymes	Metabolism	Xenobiotics biodegradation and metabolism	0.0005109	0.00476329	7
profile 13	ko04974	Protein digestion and absorption	Organismal Systems	Digestive system	0.00063096	0.00549044	7
profile 13	ko04213	Longevity regulating pathway–multiple species	Organismal Systems	Aging	0.00072624	0.00592462	6
profile 13	ko04915	Estrogen signalling pathway	Organismal Systems	Endocrine system	0.00077213	0.00592846	6
profile 13	ko00232	Caffeine metabolism	Metabolism	Biosynthesis of other secondary metabolites	0.00138265	0.00941067	3
profile 13	ko04142	Lysosome	Cellular Processes	Transport and catabolism	0.00144196	0.00941067	8
profile 13	ko00040	Pentose and glucuronate interconversions	Metabolism	Carbohydrate metabolism	0.00160978	0.01000564	5
profile 13	ko03051	Proteasome	Brite Hierarchies	Protein families: genetic information processing	0.00195145	0.01157796	5
profile 13	ko00480	Glutathione metabolism	Metabolism	Metabolism of other amino acids	0.00248591	0.01410768	5
profile 13	ko04260	Cardiac muscle contraction	Organismal Systems	Circulatory system	0.00278972	0.0145653	5
profile 13	ko04141	Protein processing in endoplasmic reticulum	Genetic Information Processing	Folding, sorting and degradation	0.00575689	0.02890098	7
profile 13	ko04911	Insulin secretion	Organismal Systems	Endocrine system	0.00717362	0.03344092	4
profile 17	ko01002	Peptidases and inhibitors	Brite Hierarchies	Protein families: metabolism	2.0414 × 10^−9^	1.934 × 1007	16
profile 17	ko04974	Protein digestion and absorption	Organismal Systems	Digestive system	3.4432 × 10^−5^	0.00103489	6
profile 17	ko04080	Neuroactive ligand–receptor interaction	Environmental Information Processing	Signaling molecules and interaction	4.3695 × 10^−5^	0.00103489	6
profile 17	ko04972	Pancreatic secretion	Organismal Systems	Digestive system	6.5347 × 10^−5^	0.00123816	6
profile 18	ko04812	Cytoskeleton proteins	Brite Hierarchies	Protein families: signalling and cellular processes	2.5662 × 10^−5^	0.00453818	14
profile 19	ko00537	Glycosylphosphatidylinositol (GPI)-anchored proteins	Brite Hierarchies	Protein families: signalling and cellular processes	3.0408 × 10^−6^	0.00060416	9
profile 19	ko00980	Metabolism of xenobiotics by cytochrome P450	Metabolism	Xenobiotics biodegradation and metabolism	1.0063 × 10^−5^	0.00080858	10
profile 19	ko00053	Ascorbate and aldarate metabolism	Metabolism	Carbohydrate metabolism	2.2194 × 10^−5^	0.00133745	9
profile 19	ko00480	Glutathione metabolism	Metabolism	Metabolism of other amino acids	0.00013626	0.00656902	10
profile 19	ko00982	Drug metabolism–cytochrome P450	Metabolism	Xenobiotics biodegradation and metabolism	0.00017653	0.00709216	8
profile 19	ko00730	Thiamine metabolism	Metabolism	Metabolism of cofactors and vitamins	0.00027103	0.00816646	5
profile 19	ko00910	Nitrogen metabolism	Metabolism	Energy metabolism	0.00027103	0.00816646	5
profile 19	ko04142	Lysosome	Cellular Processes	Transport and catabolism	0.00043752	0.01076957	15
profile 19	ko00620	Pyruvate metabolism	Metabolism	Carbohydrate metabolism	0.00044677	0.01076957	8
profile 19	ko00100	Steroid biosynthesis	Metabolism	Lipid metabolism	0.00056685	0.01138988	5
profile 19	ko03320	PPAR signalling pathway	Organismal Systems	Endocrine system	0.00056701	0.01138988	8
profile 19	ko00350	Tyrosine metabolism	Metabolism	Amino acid metabolism	0.00086773	0.01480567	5
profile 19	ko00983	Drug metabolism–other enzymes	Metabolism	Xenobiotics biodegradation and metabolism	0.000951	0.01480567	11
profile 19	ko00561	Glycerolipid metabolism	Metabolism	Lipid metabolism	0.00095309	0.01480567	9
profile 19	ko04972	Pancreatic secretion	Organismal Systems	Digestive system	0.00098273	0.01480567	12
profile 19	ko01002	Peptidases and inhibitors	Brite Hierarchies	Protein families: metabolism	0.00164678	0.0233506	28
profile 19	ko00071	Fatty acid degradation	Metabolism	Lipid metabolism	0.00239315	0.03204855	6
profile 19	ko00981	Insect hormone biosynthesis	Metabolism	Metabolism of terpenoids and polyketides	0.00288396	0.03658879	5
profile 19	ko04260	Cardiac muscle contraction	Organismal Systems	Circulatory system	0.00326238	0.03821649	8
profile 19	ko00410	beta-Alanine metabolism	Metabolism	Metabolism of other amino acids	0.00332934	0.03821649	5
profile 19	ko00380	Tryptophan metabolism	Metabolism	Amino acid metabolism	0.00382213	0.04187888	5

## Data Availability

RNA-seq data were deposited in the Sequence Read Archive (SRA) and are accessible under accession number PRJNA833604.

## Supplementary Materials

The following supporting information can be downloaded from https://www.mdpi.com/article/10.3390/ijms232012322/s1.
